# Pervasive Divergence of Transcriptional Gene Regulation in Caenorhabditis Nematodes

**DOI:** 10.1371/journal.pgen.1004435

**Published:** 2014-06-26

**Authors:** Antoine Barrière, Ilya Ruvinsky

**Affiliations:** 1 Department of Ecology and Evolution and Institute for Genomics and Systems Biology, The University of Chicago, Chicago, Illinois, United States of America; 2 Department of Organismal Biology and Anatomy, The University of Chicago, Chicago, Illinois, United States of America; University of Washington, United States of America

## Abstract

Because there is considerable variation in gene expression even between closely related species, it is clear that gene regulatory mechanisms evolve relatively rapidly. Because primary sequence conservation is an unreliable proxy for functional conservation of *cis*-regulatory elements, their assessment must be carried out *in vivo*. We conducted a survey of *cis*-regulatory conservation between *C. elegans* and closely related species *C. briggsae*, *C. remanei*, *C. brenneri*, and *C. japonica*. We tested enhancers of eight genes from these species by introducing them into *C. elegans* and analyzing the expression patterns they drove. Our results support several notable conclusions. Most exogenous *cis* elements direct expression in the same cells as their *C. elegans* orthologs, confirming gross conservation of regulatory mechanisms. However, the majority of exogenous elements, when placed in *C. elegans*, also directed expression in cells outside endogenous patterns, suggesting functional divergence. Recurrent ectopic expression of different promoters in the same *C. elegans* cells may reflect biases in the directions in which expression patterns can evolve due to shared regulatory logic of coexpressed genes. The fact that, despite differences between individual genes, several patterns repeatedly emerged from our survey, encourages us to think that general rules governing regulatory evolution may exist and be discoverable.

## Author Summary

Given the importance of gene expression changes in evolution, a better understanding of how they accumulate is desirable. However, gene regulation is a complex biochemical process and it is not *a priori* clear whether general trends even exist. We systematically addressed this question by testing, in *C. elegans*, the functions of regulatory elements of eight different genes from four other nematodes. We saw rampant variation in gene regulatory mechanisms, even between closely related species. While the differences were usually seen in a relatively small number of cells, there was a discernible trend – there were many more instances of gain, rather than loss of expression, compared to patterns directed by the *C. elegans cis* elements. Finally, the recurrence of ectopic expression in the same cells suggests that the paths open to evolution may be constrained by the composition of regulatory elements. We view these patterns as a reflection of general mechanisms of gene regulatory evolution and suggest that these can be refined, and others discovered, using systematic functional tests.

## Introduction

A complex network of molecular interactions that orchestrates gene expression provides multiple sources for regulatory variation between species [1]. Changes in transcriptional regulation can occur in two fundamentally different ways: in *trans* regulators [2], [3], for example through changes in protein sequences or expression patterns of transcription factors, or in *cis* elements via changes in identity or location of transcription factor binding sites [4], [5]. Although the importance of variation in gene regulation for evolution is well appreciated [6]–[8], many details remain to be elucidated. For example, do mutations in *cis* arise and go to fixation more frequently than changes in *trans* [9], [10]? Are regulatory mutations pleiotropic and, if so, what are their effects [11]? Our research has focused on *cis*-regulatory elements (CREs). These sequences consist of multiple transcription factor binding sites and a core promoter, but these motifs tend to be short, diffuse, and flexible in their locations [12]. Traditional sequence alignments may not therefore be reliable indicators of functional conservation [13]. Because *cis* elements integrate signals from multiple *trans*-acting factors in the context of an intact cell, their functions have to be assessed *in vivo* [14].

The study of functional evolution of *cis*-regulatory elements has relied on two approaches. One typically starts with the knowledge of the location of binding sites in a regulatory sequence of one species and is followed up by the functional tests of these binding sites in the original and other species [15], [16]. This approach is labor-intensive and is more difficult to scale. An alternative consists of assessing the functions of orthologous regulatory sequences, without detailed knowledge of identity and location of binding sites, from multiple species in the same *trans*-regulatory environment (reviewed in [17]). This approach has the advantage of being applicable to less well-studied regulatory regions and can be scaled up to multiple genes, allowing researchers to infer general rules of regulatory sequence evolution.

Because they often use different methodologies and criteria for comparisons, studies that investigate the regulatory evolution of individual genes are not easily comparable. It has therefore been difficult to generalize results and infer common features of *cis*-regulatory evolution. Still, several trends are evident. Multiple studies documented divergence [18]–[22] and constraint [23]–[25] in *cis*-regulatory mechanisms between species. While functionally equivalent enhancers in different species are often found in similar locations [26], [27], this is not always the case [28]–[30]. In some cases, differences in *cis*-regulatory mechanisms reflect divergence in endogenous expression patterns [30], [31]. In others, divergent regulatory mechanisms underlie overtly conserved endogenous expression patterns [32]–[34], suggesting compensatory changes in *cis* and in *trans* [17], [22], [35].

In this study, we aimed to survey the amount of functional variation that exists in gene regulatory elements of closely related species. *C. elegans* offers an attractive model system for this work because of its simple and invariant anatomy [36], [37], which is conserved with close relatives [38]. The ease of describing gene expression with a single-cell resolution permits more precise comparisons than those possible in other multicellular model systems. *Cis*-regulatory sequences are often located within 1 kb upstream of the translation start site [39]. Several species from the Caenorhabditis genus that are approximately as divergent as human and mouse [40] are routinely used for comparisons with *C. elegans*.

We selected eight genes from five Caenorhabditis species that have available genome sequences: *C. elegans*, *C. briggsae*, *C. remanei*, and *C. brenneri*, the latter three equidistant to *C. elegans*; and *C. japonica*, a more distantly related species. In all cases, orthologous regulatory sequences were cloned, and the expression patterns they drove were evaluated in the *C. elegans trans*-regulatory environment. We report several general trends of *cis*-regulatory divergence gleaned from these observations.

## Results

### Rationale and approach

The goal of this study is essentially comparative, that is, to test whether orthologous *cis* elements are functionally equivalent. Our work is part of a broader research program aiming to investigate functional divergence of gene regulatory systems [41]. In this study we introduced *cis*-regulatory sequences (fused to GFP reporters) from several Caenorhabditis species into *C. elegans* and compared their expression patterns to those of their *C. elegans* orthologs. This approach can be seen as an extension of a fruitful paradigm that analyzes gene expression in hybrid organisms [21], [42]–[44]. In our experiments the “hybrid” portions of the genome range from a few hundred to a few thousand nucleotides directing gene expression.

While it is certainly desirable to document endogenous gene expression patterns and uncover all regulatory elements required to direct them, these questions remain outside the scope of our experimental program. Instead, our goal is to assess functional conservation of *cis*-regulatory sequences. To do so, we need only to ascertain whether *cis* elements from different species direct the same or different expression patterns. To ensure comparability, only the sequences from the immediately upstream regions were considered; consequently, if some regulatory sequences are located in introns, transgenes may not recapitulate the entire endogenous expression patterns. Movements of *cis* elements between the upstream intergenic regions in one species and introns in another, dubbed “nomadic” enhancers [30], illustrate one type of regulatory divergence our approach can uncover. Due to the persistence of the GFP protein, we are unlikely to detect minor dynamic differences in expression patterns. Testing all *cis*-regulatory elements in the common *trans*-regulatory environment of *C. elegans* simplifies the interpretation of these comparative data – any difference in expression patterns, whether gain or loss, reveals functional divergence between orthologous *cis*-regulatory elements, regardless of the expression patterns driven by these sequences in their endogenous *trans*-regulatory environments.

### Selection of species and genes to be tested

**Figure 1:**
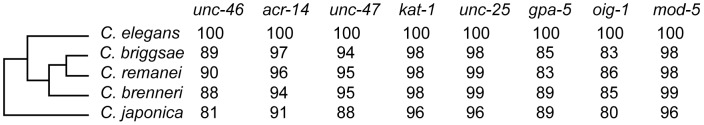
Species and genes included in this study. Phylogenetic relationship of the five studied species. Numbers represent relative conservation of protein sequences (compared to *C. elegans*) based on the BLOSUM matrix.

In addition to *C. elegans*, we selected for our study four species with sequenced genomes: *C. briggsae*, *C. remanei*, *C brenneri*, and *C. japonica* [45], [46]. We decided to focus on these species because, based on previous experience [19], [22], [47]–[51], we anticipated many *cis*-regulatory functions to be substantially conserved. Given the established phylogenetic relationships between these five species [52], our experiments interrogated the extent of functional divergence accumulated over two time scales – one between *C. elegans* and the equidistant *C. briggsae/C. remanei/C brenneri*, and another between *C. elegans* and a more distant *C. japonica* (Figure 1). Estimates suggest that the phylogenetic distance between the latter pair of species is comparable to that within the Sophophora subgenus of Drosophila [40], [52] or vertebrate classes [53]. While the phylogeny is well-resolved, the paucity of fossil Rhabditidae nematodes [54] precludes a reliable estimate of the age of species divergence.

We focused on eight genes expressed in relatively small groups of easily identifiable cells. Three genes are terminal effectors of the GABAergic fate: *unc-25* [55], *unc-46* [56], *unc-47* [57], and are thus expressed in all GABAergic neurons. Two other genes, *oig-1* and *acr-14* [58], are thought to be expressed in subsets of GABAergic neurons. We chose these five coexpressed and partially coregulated [58] genes to test whether shared regulation imposes particular constraints on their evolution. To offset this bias to a particular class of neurons, we added two genes expressed in other neuronal types – one expressed in amphid (chemosensory) neurons, *gpa-5* [59], and one expressed in serotonergic neurons, *mod-5* [60]. The pattern of serotonergic neurons is conserved between *C. elegans*, *C. briggsae* and *C. remanei* [61]; the pattern of GABAergic neurons is conserved between *C. elegans* and *C. briggsae* [22], as well as with *C. remanei* and *C. brenneri* (AB & IR, unpublished data). Finally, we included one gene expressed outside the nervous system, *kat-1*, which encodes a conserved thiolase [62] involved in a fat storage pathway [63].

The protein-coding sequences of all eight genes are highly conserved (Figure 1). Moreover, the synteny with the immediate upstream genes is conserved among all five species (Figure S1), making us confident that all of them are single-copy, one-to-one orthologs of the *C. elegans* genes. We tested the entire intergenic regions containing putative *cis* elements to ensure that comparisons indeed included orthologous regulatory sequences.

**Figure 2:**
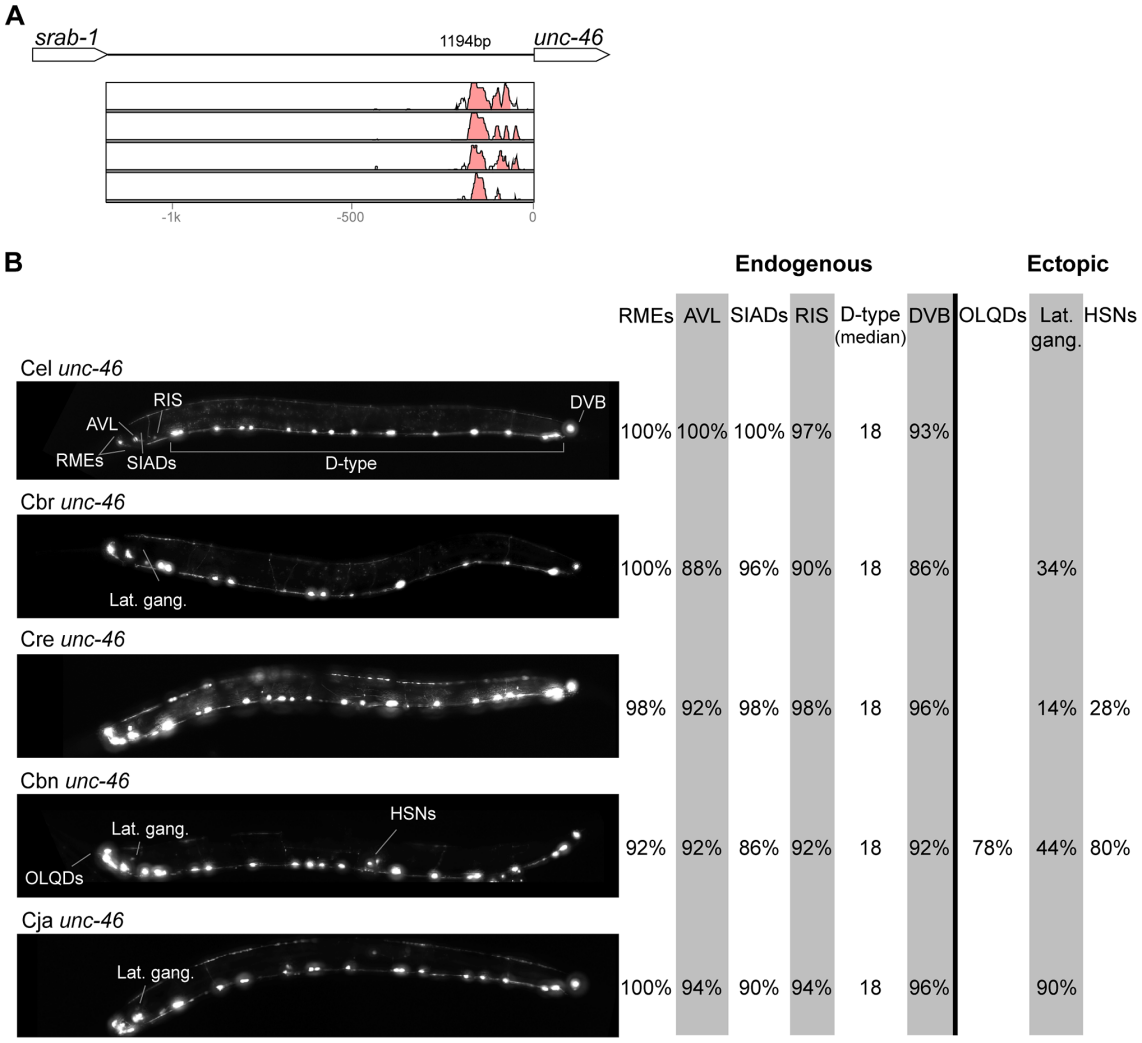
Functional conservation and divergence of *unc-46* regulation. (A) Vista plots represent primary sequence conservation in the intergenic region upstream of *unc-46*, relative to *C. elegans*. Window size = 20 bp, threshold: 70%. From top to bottom: *C. briggsae*, *C. remanei*, *C. brenneri*, *C. japonica*. (B) Expression patterns driven by the *C. elegans* (Cel), *C. briggsae* (Cbr), *C. remanei* (Cre), *C. brenneri* (Cbn), and *C. japonica* (Cja) CREs of *unc-46*. For all cells, frequency of expression is indicated, except for D-type neurons for which the median number of expressing cells in shown. For groups of multiple cells, percentages represent frequency of expression in at least one of these cells: RMEs(RMED/V/L/R), SIADs (SIADL/R), OLQDs (OLQDL/R), Lat. gang. (unidentified pair of neurons in the lateral ganglion), HSNs (HSNL/R). Detailed data are shown in Table S3.

**Figure 3:**
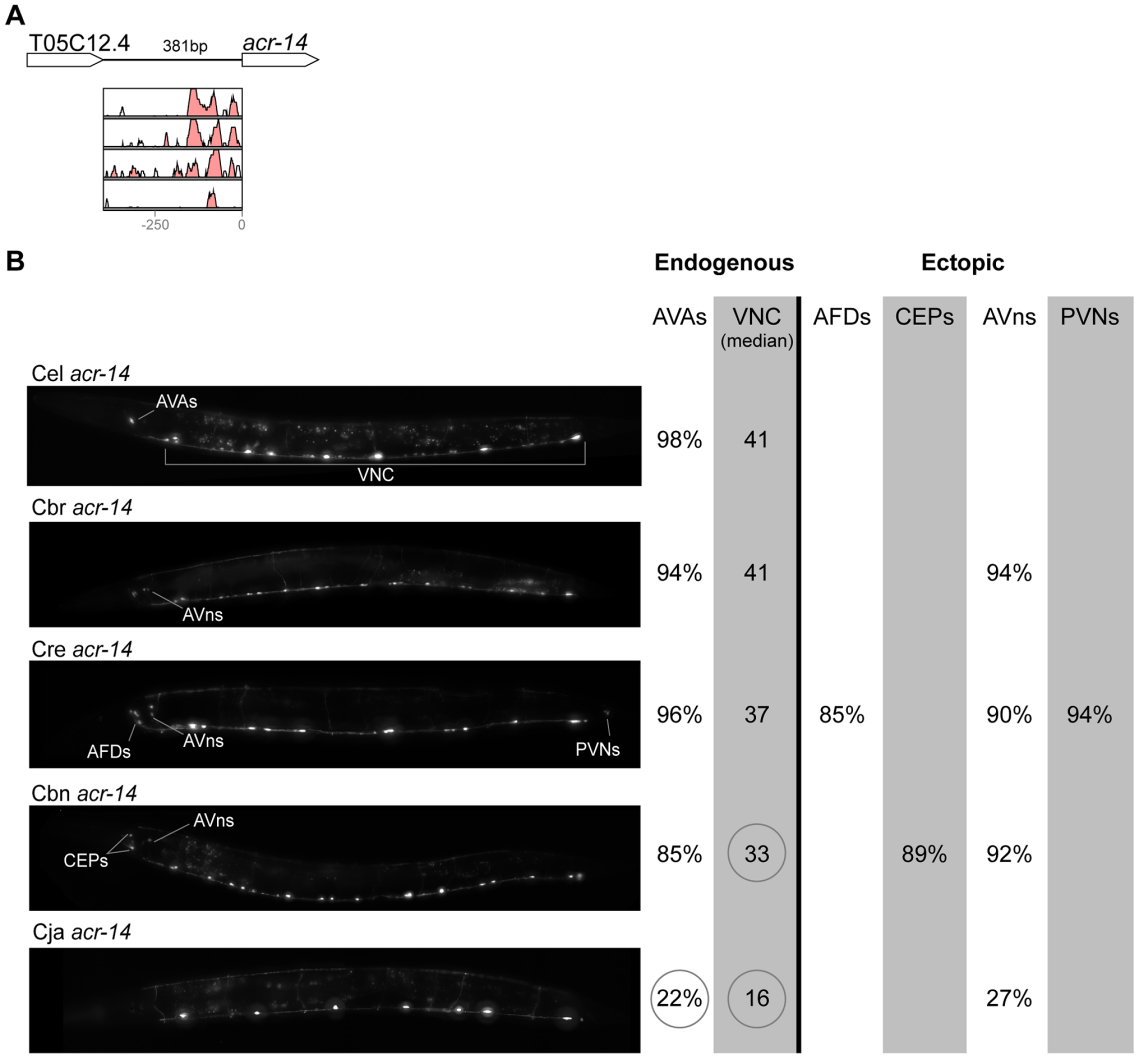
Functional conservation and divergence of *acr-14* regulation. (A) Vista plots represent primary sequence conservation in the intergenic region upstream of *acr-14*, relative to *C. elegans*. Window size = 20 bp, threshold: 70%. From top to bottom: *C. briggsae*, *C. remanei*, *C. brenneri*, *C. japonica*. (B) Expression patterns driven by the *C. elegans* (Cel), *C. briggsae* (Cbr), *C. remanei* (Cre), *C. brenneri* (Cbn), and *C. japonica* (Cja) CREs of *acr-14*. For all cells, frequency of expression is indicated, except for the ventral nerve cord (VNC) for which the median number of expressing cells in shown. For groups of multiple cells, percentages represent frequency of expression in at least one of these cells: AVAs (AVAL/R), AFDs (AFDL/R), CEPs (CEPD/V L/R), AVns (AVHL/R or AVJL/R or AVDL/R), PVNs (PVNL/R). Reductions of expression compared to the endogenous pattern are circled. Detailed data are shown in Table S4.

**Figure 4:**
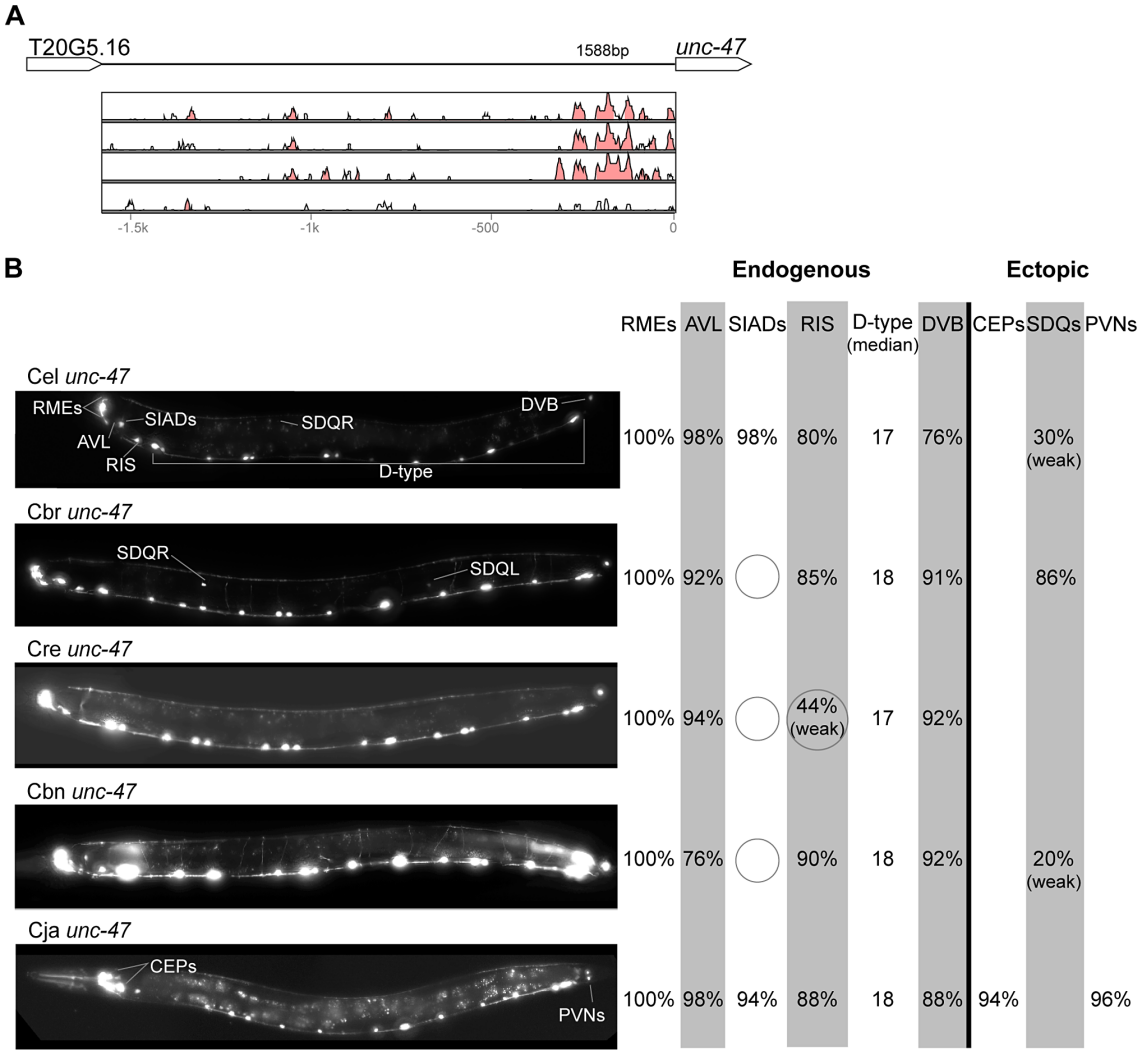
Functional conservation and divergence of *unc-47* regulation. (A) Vista plots represent primary sequence conservation in the intergenic region upstream of *unc-47*, relative to *C. elegans*. Window size = 20 bp, threshold: 70%. From top to bottom: *C. briggsae*, *C. remanei*, *C. brenneri*, *C. japonica*. (B) Expression patterns driven by the *C. elegans* (Cel), *C. briggsae* (Cbr), *C. remanei* (Cre), *C. brenneri* (Cbn), and *C. japonica* (Cja) CREs of *unc-47*. For all cells, frequency of expression is indicated, except for D-type neurons for which the median number of expressing cells in shown. For groups of multiple cells, percentages represent frequency of expression in at least one of these cells: RMEs (RMED/V/L/R), SIADs (SIADL/R), CEPs (CEPD/V L/R), SDQs (SDQL/R), PVNs (PVNL/R). It is unclear whether expression in the SIADs is endogenous [56], [57], [68]. However, since it is consistently seen with the *C. elegans* CRE, we included it in the endogenous pattern. We classified the strong expression of the *C. briggsae unc-47* CRE in SDQL/R as ectopic, even though weak SDQR expression was observed with the *C. elegans* CRE, because of the dramatic differences in the frequency and intensity of expression [22]. Reduction and losses of expression compared to the endogenous pattern are circled. Detailed data are shown in Table S5.

**Figure 5:**
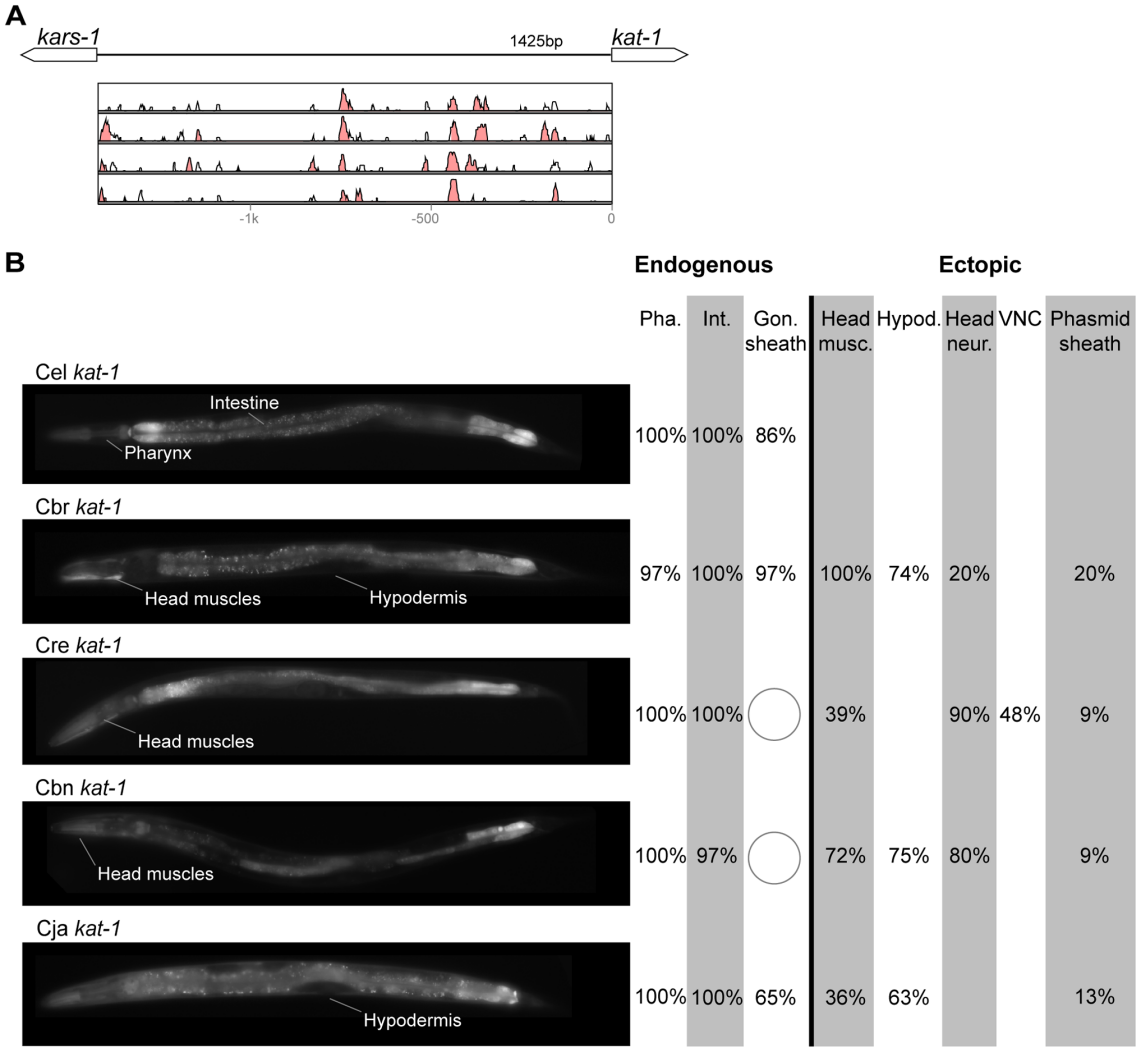
Functional conservation and divergence of *kat-1* regulation. (A) Vista plots represent primary sequence conservation in the intergenic region upstream of *kat-1*, relative to *C. elegans*. Window size = 20 bp, threshold: 70%. From top to bottom: *C. briggsae*, *C. remanei*, *C. brenneri*, *C. japonica*. (B) Expression patterns driven by the *C. elegans* (Cel), *C. briggsae* (Cbr), *C. remanei* (Cre), *C. brenneri* (Cbn), and *C. japonica* (Cja) CREs of *kat-1*. Frequency of expression in different tissues is shown: Pha. (pharynx), Int. (intestine), Gon. sheath (gonadal sheath), Head musc. (head muscles), Hypod. (hypodermis), Head neur. (head neurons), VNC (ventral nerve cord). Losses of expression compared to the endogenous pattern are circled. Detailed data are shown in Table S6.

**Figure 6:**
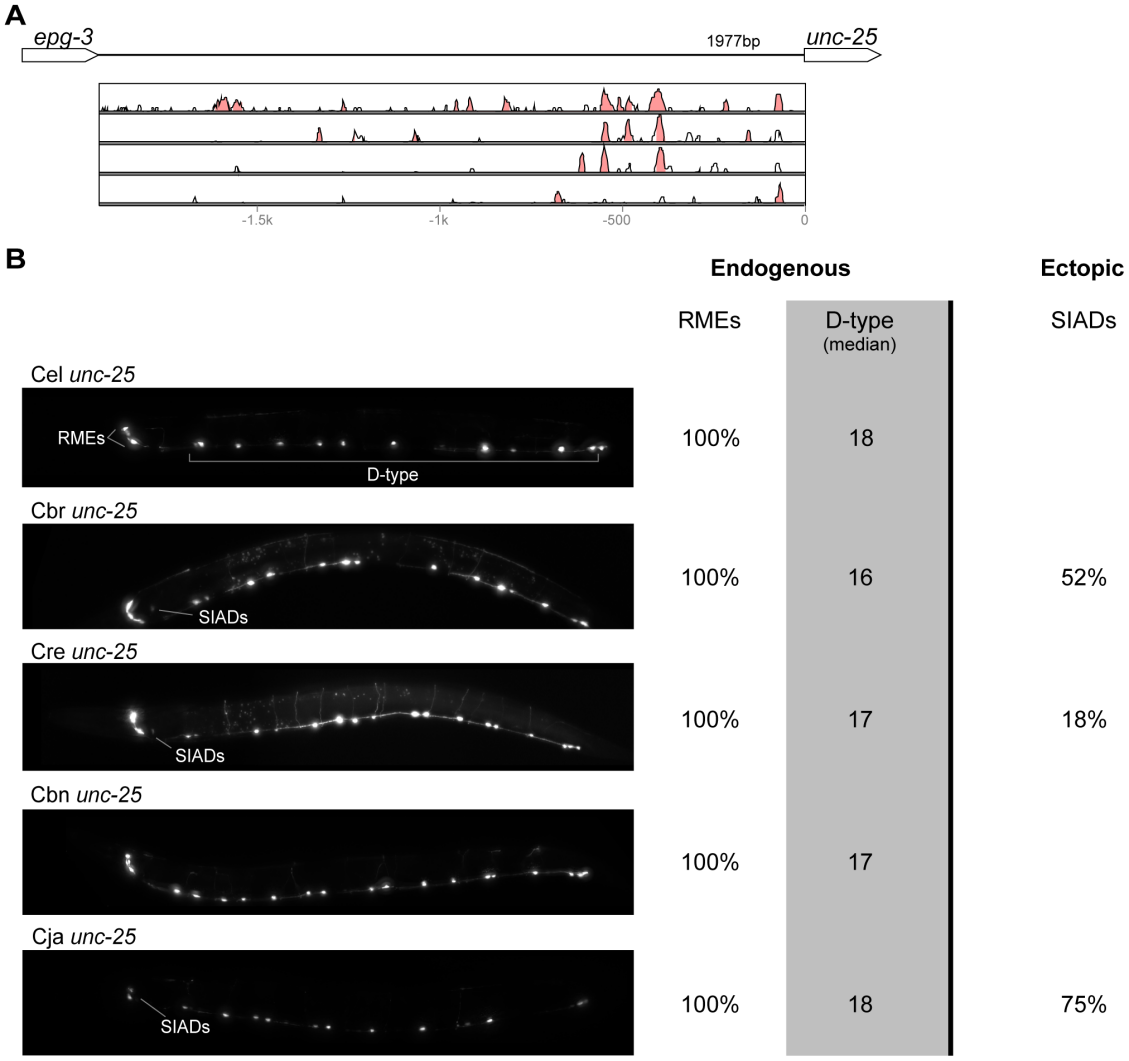
Functional conservation and divergence of *unc-25* regulation. (A) Vista plots represent primary sequence conservation in the intergenic region upstream of *unc-25*, relative to *C. elegans*. Window size = 20 bp, threshold: 70%. From top to bottom: *C. briggsae*, *C. remanei*, *C. brenneri*, *C. japonica*. (B) Expression patterns driven by the *C. elegans* (Cel), *C. briggsae* (Cbr), *C. remanei* (Cre), *C. brenneri* (Cbn), and *C. japonica* (Cja) CREs of *unc-25*. For all cells, frequency of expression is indicated, except for D-type neurons for which the median number of expressing cells in shown. For groups of multiple cells, percentages represent frequency of expression in at least one of these cells: RMEs (RMED/V/L/R), SIADs (SIADL/R). Detailed data are shown in Table S7.

**Figure 7:**
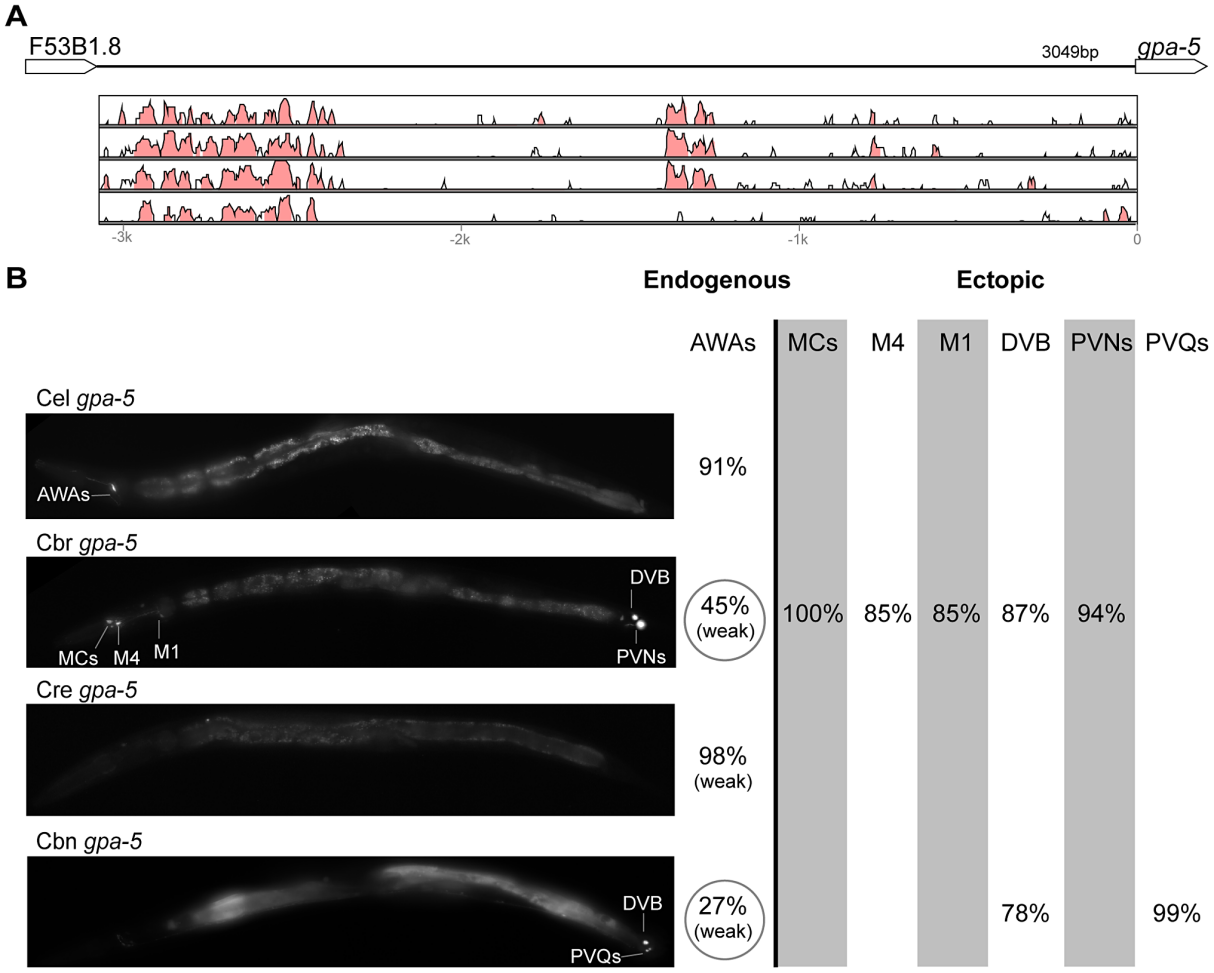
Functional conservation and divergence of *gpa-5* regulation. (A) Vista plots represent primary sequence conservation in the intergenic region upstream of *gpa-5*, relative to *C. elegans*. Window size = 20 bp, threshold: 70%. From top to bottom: *C. briggsae*, *C. remanei*, *C. brenneri*, *C. japonica*. (B) Expression patterns driven by the *C. elegans* (Cel), *C. briggsae* (Cbr), *C. remanei* (Cre), and *C. brenneri* (Cbn) CREs of *gpa-5*. For all cells, frequency of expression is indicated. For groups of multiple cells, percentages represent frequency of expression in at least one of these cells: AWAs (AWAL/R), MCs (MCL/R), PVNs (PVNL/R), PVQs (PVQL/R). Reductions of expression compared to the endogenous pattern are circled. Detailed data are shown in Table S8.

**Figure 8:**
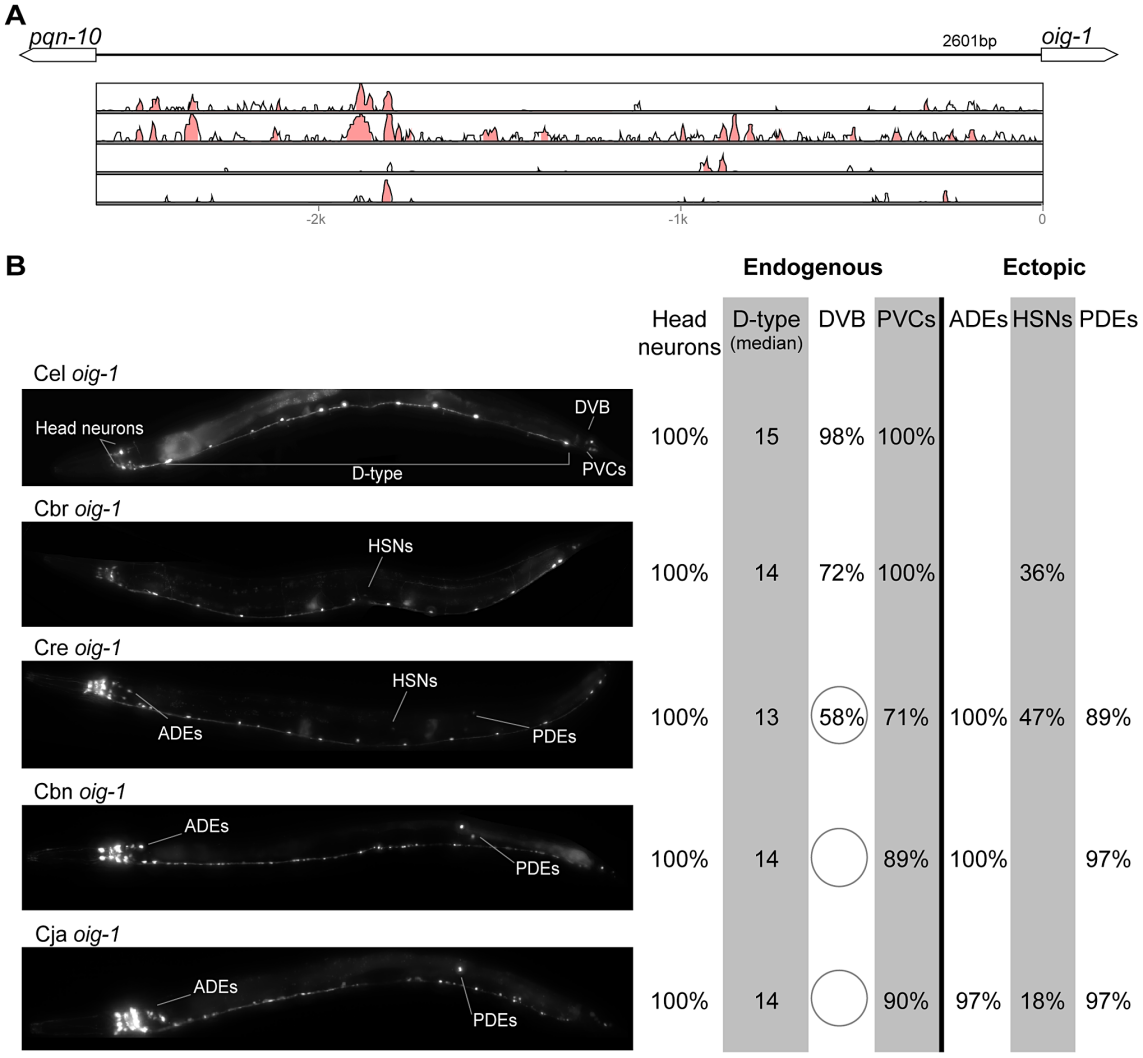
Functional conservation and divergence of *oig-1* regulation. (A) Vista plots represent primary sequence conservation in the intergenic region upstream of *oig-1*, relative to *C. elegans*. Window size = 20 bp, threshold: 70%. From top to bottom: *C. briggsae*, *C. remanei*, *C. brenneri*, *C. japonica*. (B) Expression patterns driven by the *C. elegans* (Cel), *C. briggsae* (Cbr), *C. remanei* (Cre), *C. brenneri* (Cbn), and *C. japonica* (Cja) CREs of *oig-1*. For all cells, frequency of expression is indicated, except for D-type neurons for which the median number of expressing cells in shown. For groups of multiple cells, percentages represent frequency of expression in at least one of these cells: Head neurons (large cluster of head neurons, including ALAL/R, SMDVL/R, RMDVL/R, RIAL/R, AVAL/R, RIML/R, RMDDL/R and IL1s), PVCs (PVCL/R), ADEs (ADEL/R), HSNs (HSNL/R), PDEs (PDEL/R). Reductions and losses of expression compared to the endogenous pattern are circled. Detailed data are shown in Table S9.

**Figure 9:**
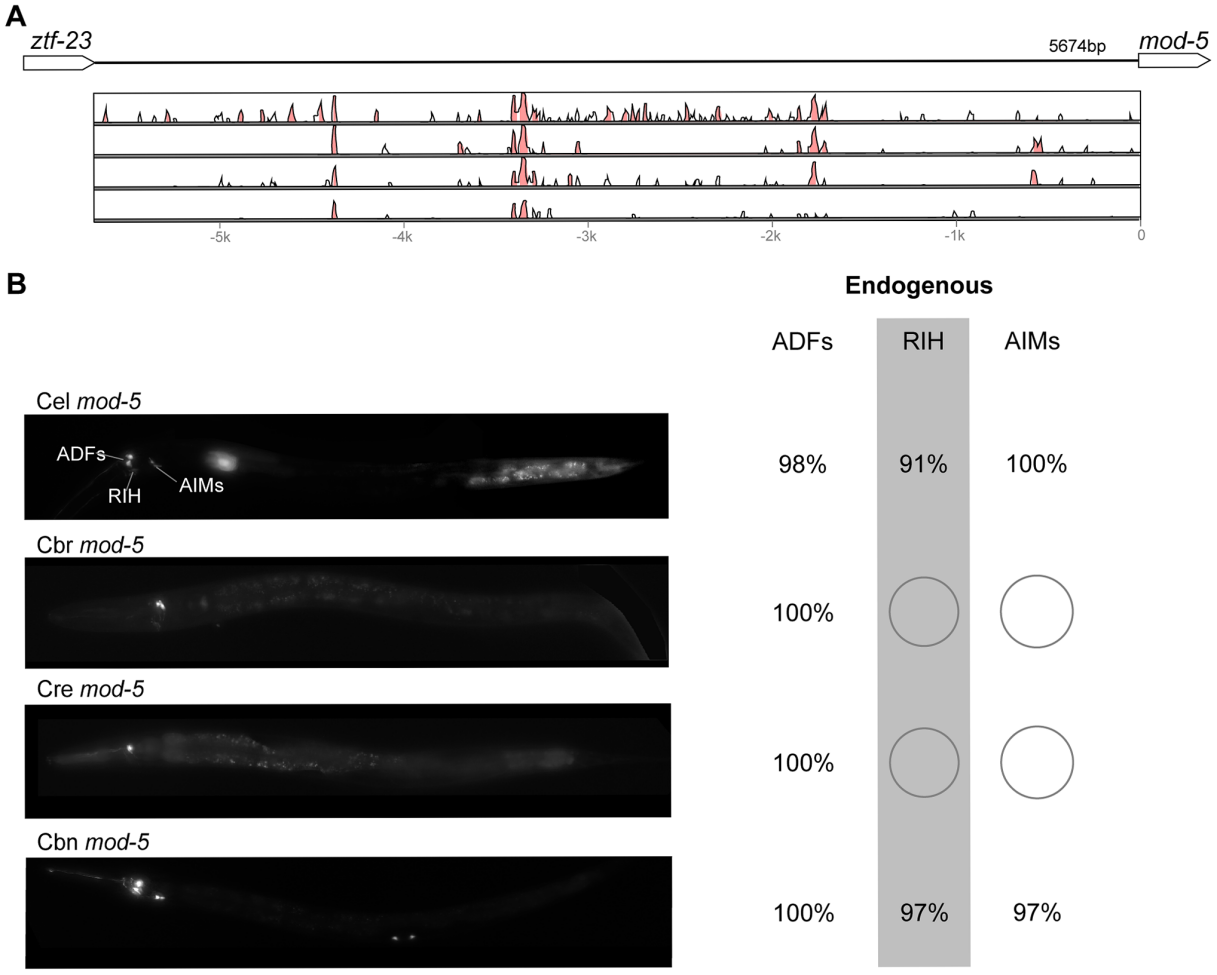
Functional conservation and divergence of *mod-5* regulation. (A) Vista plots represent primary sequence conservation in the intergenic region upstream of *mod-5*, relative to *C. elegans*. Window size = 20 bp, threshold: 70%. From top to bottom: *C. briggsae*, *C. remanei*, *C. brenneri*, *C. japonica*. (B) Expression patterns driven by the *C. elegans* (Cel), *C. briggsae* (Cbr), *C. remanei* (Cre), and *C. brenneri* (Cbn) CREs of *mod-5*. For all cells, frequency of expression is indicated. For groups of multiple cells, percentages represent frequency of expression in at least one of these cells: ADFs (ADFL/R), AIMs (AIML/R). Losses of expression compared to the endogenous pattern are circled. Detailed data are shown in Table S10.

In contrast with the high conservation of coding sequences, the noncoding upstream regions (which we assume to contain the majority of CREs [39]) are much more variable. We aligned orthologous intergenic sequences upstream of *C. briggsae*, *C. remanei*, *C. brenneri*, and *C. japonica* to their *C. elegans* counterparts and visualized the results using software package VISTA [64]. The CREs of *unc-46*, *acr-14*, and *unc-47* showed relatively high levels of conservation, spanning ∼150 to 300 nucleotides in most or all species (Figures 2A–4A). The CREs of *kat-1* and *unc-25* displayed somewhat lower conservation, although some blocks of high similarity could still be clearly identified (Figures 5A, 6A). The CREs of *gpa-*5, *oig-1*, and *mod-5* had little obvious evidence of conservation in the proximity of the translation start site (Figures 7A–9A), although some regions of putative conservation were present substantially upstream of these genes. Sequence comparisons within non-coding regions are notoriously challenging because we do not understand the “rules” by which these sequences evolve [1]. Therefore, we considered two additional measures of sequence divergence, namely the length of the longest contiguous sequence that is perfectly conserved between orthologs and the number of nucleotides contained within blocks of perfect conservation of 7 bp and longer. By both of these measures, *cis* elements of *unc-46* and *acr-14*, and to some extent *unc-47*, appear to be more conserved than those of the rest of the genes included in this study (Table S1). Next we tested functional conservation of these regulatory elements. In all experiments we used sequences upstream of translation start sites, thus making translational fusion genes, to ensure that the tested regions encompass basal promoters and more distal regulatory sequences.

### Pervasive functional divergence in *cis* elements

Expression patterns directed in *C. elegans* by the orthologous *cis* elements of the eight studied genes were largely similar (Figures 2–9; detailed descriptions of the observed patterns are presented in Text S1). However, patterns driven by heterologous CREs were indistinguishable from those directed by their *C. elegans* orthologs in only three instances: *C. brenneri unc-25* (Figure 6B), *C. remanei gpa-5* (Figure 7B), and *C. brenneri mod-5* (Figure 9B). In the rest of the cases, the expression patterns of heterologous CREs differed from their *C. elegans* counterparts. Some failed to direct expression in some of the cells in which *C. elegans cis* elements were active, others drove expression in additional cells. For reasons of brevity, in the following we will refer to the former as “losses” and to the latter as “gains” or ectopic expression, without the implication that these reflect differences in endogenous expression patterns. They do, however, reveal instances of divergence of the regulatory mechanisms controlling expression of orthologous genes in the examined species.

“Losses” of expression in the endogenous pattern typically affected single cell types. In two cases (*unc-46* and *unc-25*; Figures 2B and 6B), the expression patterns driven by the *C. elegans* CREs were completely recapitulated by all heterologous CREs. In three instances (*unc-47*, *gpa-5*, and *oig-1*; Figures 4B, 7B, and 8B), while the patterns were qualitatively conserved, portions directed by one or more heterologous CREs were markedly decreased, in frequency or intensity. For example, the *C. remanei cis* element of *unc-47* drives weak and inconsistent expression in the neuron RIS (Figure 4B), the *C. briggsae* and *C. brenneri* CREs of *gpa-5* direct weak and inconsistent expression in AWAL/R (Figure 7B), and the *C. remanei*, *C. brenneri* and *C. japonica* CREs of *oig-1* are expressed inconsistently in DVB (Figure 8B). The *C. japonica* CRE of *acr-14* fails to direct expression in several cell types in the ventral nerve cord, only maintaining expression in D-type neurons, while expression in AVAL/R is much weaker than with other species' CREs (Figure 3B). The *C. remanei* and *C. brenneri* CREs of *kat-1* fail to drive expression in the gonadal sheath (Figure 5B), the somatic tissue enveloping the proximal gonad. In the most severe case, *mod-5*, the CREs from *C. briggsae* and *C. remanei* only support expression in ADFL/R (Figure 9B).

In addition to “losses” of expression in subsets of endogenous patterns, most heterologous *cis* elements also drove ectopic expression. Indeed, only six tested CREs did not show any evidence of “gain” of expression: *C. remanei unc-47* (Figure 4B), *C. brenneri unc-25* (Figure 6B), *C. remanei gpa-5* (Figure 7B), and all three heterologous *cis* elements of *mod-5* (Figure 9B). Ectopic expression was seen in as few as one and as many as five different cell types, depending on the gene. In some cases, this expression was driven in the same cells or tissues by all heterologous CREs of a given gene: unidentified lateral ganglion neurons in the head (*unc-46*, Figure 2B), AVnL/R neurons in the lateral ganglion (*acr-14*, Figure 3B), and head muscles (*kat-*1, Figure 5B). In other instances, only some of the orthologous elements directed co-occurring expression: HSNL/R for *unc-46* (Figure 2B), hypodermis for *kat-1* (Figure 5B), DVB for *gpa-5* (Figure 7B), and ADEL/R, PDEL/R, HSNL/R with *oig-1* (Figure 8B).

The results described above reveal pervasive divergence in *cis*-regulatory function. However, divergence can also stem from changes in *trans* regulators [2], [3]. To test whether the *trans* environments were functionally equivalent between species, we compared spatial expression patterns driven by four *C. briggsae* CREs in *C. elegans* and *C. briggsae*. Although expression patterns generated by these sequences were qualitatively similar between the two species, in every instance there were reproducible differences as well (Figure S2). These results further reinforce the notion that divergence has taken place in both *cis*- and *trans*-regulatory mechanisms.

## Discussion

We carried out functional comparisons of orthologous regulatory elements of eight genes from Caenorhabditis nematodes. Our experimental paradigm, placing orthologous *cis* elements into the common *trans*-regulatory environment of *C. elegans*, allows inferences to be made about the extent of functional divergence between *C. elegans* CREs and their orthologs from other species. Because we selected genes expressed in relatively simple patterns, we were able to detect even subtle differences. Our results support four notable conclusions.

### Divergence is pervasive

Most of the orthologous *cis* elements we analyzed directed patterns of expression in *C. elegans* that either substantially or completely matched the expression patterns of the orthologous *C. elegans* CREs (Figures 2–8; with the possible exception of *mod-5*, Figure 9). This result, supported by 30 transgenes, suggests that the mechanisms controlling orthologous gene expression are largely conserved among the studied species. Yet, in the vast majority of these cases (27/30), orthologous CREs directed expression patterns that differed from their *C. elegans* counterparts. These differences were fairly subtle, typically affecting only a few cells, as previously reported in other species [65]–[67] highlighting the value of detailed, focused, multi-gene analyses to reveal trends. Differences in the lengths of tested *cis* elements did not appear to correlate with the observed differences in expression patterns (Figure S3).

We observed “losses”, as well as “gains” of expression, as compared to the patterns generated by the *C. elegans* CREs. Even *cis* elements from two closely related species, *C. briggsae* and *C. remanei*, often differed in the expression patterns they directed, indicating that divergence could accumulate relatively quickly. Because in most instances it is difficult to establish the precise endogenous expression patterns of the genes, the observed differences either reflect lineage-specific changes in gene expression or divergence in the mechanisms that regulate conserved expression. In several cases, however, compelling indirect evidence points to the latter scenario.

Three of the eight genes in this study, *unc-25*, *unc-46*, and *unc-47*, are terminal effectors of the GABAergic neuronal fate. Immunostaining for GABA in *C. elegans* [68], *Ascaris suum* [69], and *C. briggsae* and *C. remanei* (AB & IR, unpublished data) revealed very similar patterns. Furthermore, the expression driven by the *C. briggsae unc-47* CRE in its endogenous *trans*-regulatory environment is identical to that driven by the *C. elegans unc-47* CRE in *C. elegans* [22]. Similarly, patterns of immunostaining for serotonin in *C. elegans*, *C. briggsae*, and *C. remanei* were identical [61], [70]. These results suggest that the number and relative position of GABAergic and serotonergic neurons, and thus the expression patterns of key genes defining these neuronal fates (the three GABA genes above and *mod-5*), are conserved among these Caenorhabditis nematodes. Thus, differences in *cis* regulatory elements of these four genes (Figures 2B, 4B, 6B, 9B) likely reveal changes in the specific ways in which these conserved expression patterns are encoded. This interpretation stresses noticeable divergence in gene regulation even between closely related lineages, consistent with what has been seen in others species [71], [72]. This view suggests that changes in *trans*-regulatory mechanisms and *cis*-regulatory elements accumulate in a somewhat compensatory fashion to ensure that the overall expression patterns of genes remain conserved [22], [35], [42], [73]. The different expression patterns of four *C. briggsae* CREs in *C. elegans* and *C. briggsae* (Figure S2) support the idea that *trans*-regulatory divergence is prevalent.

### Sequence conservation and functional divergence

Consistent with previous reports [73]–[75], we saw no obvious correspondence between the extent of large-scale sequence conservation and functional conservation. For example, while the CREs of *unc-25* and *oig-1* show relatively scant primary sequence conservation, their functions appear to be conserved no less well (Figures 6, 8) than those of genes with apparently greater sequence conservation (e.g. *unc-46*, Figure 2). Sequence comparisons in noncoding regions, particularly when these are of different length, are notoriously challenging. Other metrics of sequence similarity, like the portion of the CRE that is conserved, also failed to reveal a discernible relationship to functional conservation (Figure S3, Table S1). We also tested shorter *cis* elements of *mod-5* and *unc-25* that excluded the majority of conserved sequence blocks; their expression patterns were qualitatively similar to those of their longer counterparts (data not shown). These findings are consistent with previous reports that conserved expression patterns can be driven by highly divergent regulatory elements [76]–[83]. Previous research suggested that at least in some instances, long tracts of conserved sequences in *cis* elements may reflect particular features of regulatory organization, rather than unusually stringent selection for the maintenance of expression patterns [84].

Collectively, these results suggest that we may need to reevaluate a common reliance on large-scale sequence conservation when using comparative sequence data to identify *cis*-regulatory elements. Presence or absence of transcription factors binding sites, their arrangement and spacing may be more informative, although harder to detect [73], [85]–[89].

We did not detect greater functional divergence of CREs from the more distant *C. japonica* compared to *C. briggsae*, *C. remanei*, and *C. brenneri*. Among the six genes that have been tested from all four of these species, *C. japonica cis* elements show approximately the same number of “gains” and “losses” as their orthologs from other species (Table S2). It is possible that the ∼2-fold difference in the phylogenetic distance [40] separating, on the one hand, *C. elegans* and *C. japonica* and, on the other hand, *C. elegans* and *C. briggsae*/*C. remanei*/*C. brenneri*, does not offer enough power to test this hypothesis. Examining more distantly related pairs of species may be required. Finally, the complexity of the expression pattern of a gene does not seem to be correlated with the amount of functional divergence in its *cis* element (Figure S3).

### “Gains” are more common than “losses”

One striking pattern evident in our results is that a substantial majority of functional differences between orthologous *cis* elements is due to “gain”, rather than “loss” or reduction, of expression relative to the pattern directed by the *C. elegans* CREs. Put another way, when tested in *C. elegans*, heterologous regulatory elements more commonly directed expression in more rather than fewer cells, compared to the *C. elegans*-driven patterns. When all experiments reported here are considered together, the total number of “gains” was nearly three-fold higher than the number of “losses” (51 vs. 18). Even when minor differences in patterns are counted as “losses”, their number (23) is still less than half than that of “gains” (51). This phenomenon does not appear to be due to greater power to detect “gains” compared to “losses” (Figure S4). Restricting comparisons only to those genes for which all four non-*C. elegans* species were tested, does not substantially alter this conclusion (12 vs. 44 or 16 vs. 44, if “losses” are counted more liberally). Therefore, our results suggest that the two regulatory modalities, namely one directing expression in certain cells and another repressing inappropriate expression, evolve at different rates. The molecular mechanisms and evolutionary forces that could account for this observation remain to be investigated. It is possible, however, that the positive and negative regulatory aspects of gene regulation evolve under different regimes, because of the difference in the ways in which they are encoded within *cis* elements.

### Recurrent divergence patterns suggest developmental bias in evolutionary trajectories

**Figure 10:**
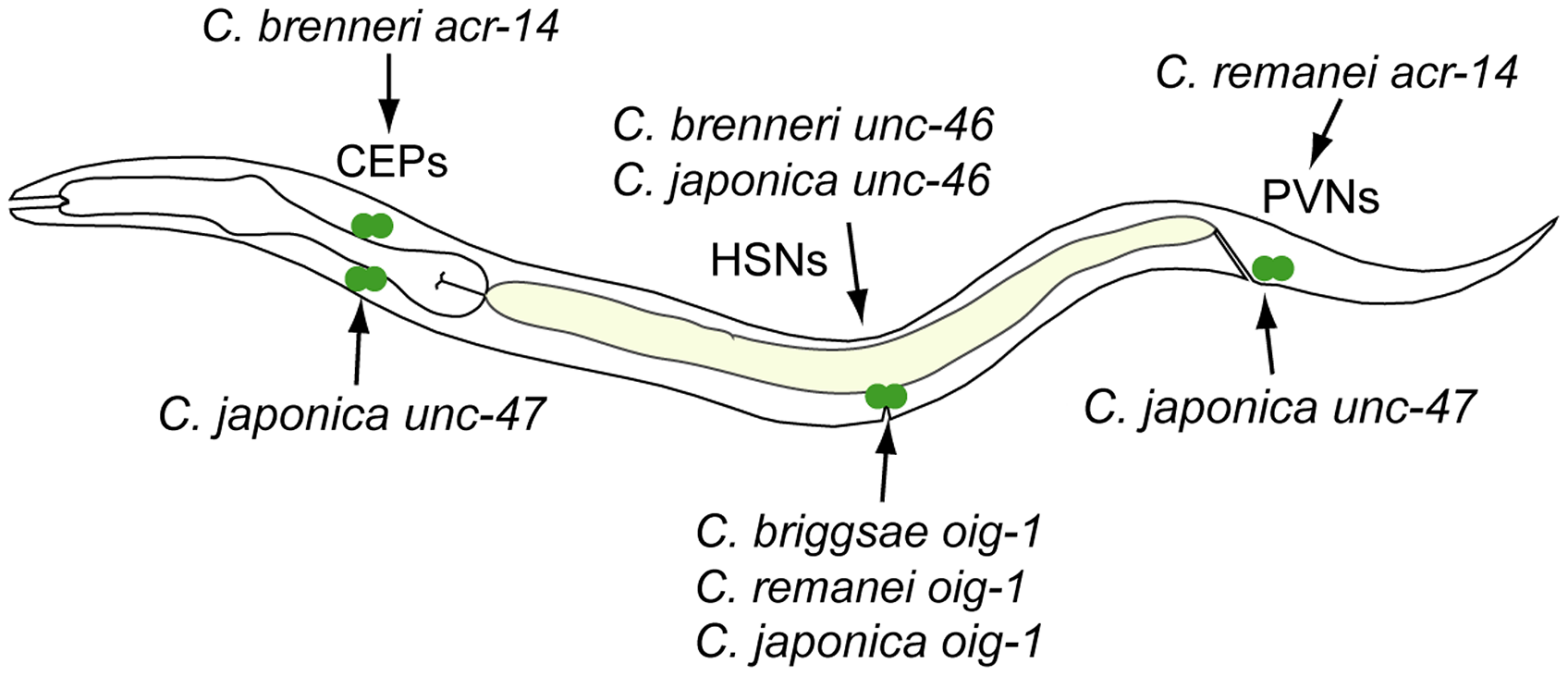
Recurrent “gains” of expression by different CREs. CREs of *acr-14* from *C. brenneri* (Figure 3B) and *unc-47* from *C. japonica* (Figure 4B) drive expression in CEP neurons in the head. CREs of *unc-46* from *C. brenneri* and *C. japonica* (Figure 2B) and *oig-1* from *C. briggsae*, *C. remanei* and *C. brenneri* (Figure 8B) drive expression in HSN neurons in the mid-body. CREs of *acr-14* from *C. remanei* (Figure 3B) and *unc-47* from *C. japonica* (Figure 4B) drive expression in PVN neurons in the tail.

The relatively large number of cases in which heterologous *cis* elements directed ectopic expression when in C. elegans, allowed us to investigate whether these “gains” followed a pattern. Notably, for the neuronal genes *unc-46*, *acr-14*, *unc-47*, *unc-25*, *oig-1*, and *gpa-5*, nearly all “gains” occurred in neurons (Figures 2–4, 6–8). This tropism suggests that the regulatory architecture of neuronal CREs – some transcriptional inputs are pan-neuronal in nature [90], [91] – may restrict ectopic expression to neurons. We further noted that in several instances, CREs of different genes or from different species directed ectopic expression in the same cells (Figure 10). The cells “gaining” expression do not appear to be transcriptionally promiscuous, because ectopic expression is seen in several different cells not previously noted for indiscriminate expression (Text S1). Furthermore, the “gain” of expression is not likely to be due to effects of vector sequences. We used a standard vector utilized by us and others thousands of times. Previous studies using this vector documented ectopic expression in the intestine and pharynx [49], [92], not specific subsets of neurons, as we reported here. Instead, we favor a hypothesis that the *cis* elements themselves could be sharing certain characteristics that make them more likely to direct expression in particular cells. The recurrent “gains” of expression were seen for *unc-46*, *acr-14*, *unc-47*, and *oig-1*, which are coexpressed in a subset of GABAergic neurons and are know to be coregulated by at least one transcription factor, UNC-30 [58]. It is therefore plausible that these *cis* elements share some features, for example transcription factor binding sites or general organization, and that this similarity may bias the trajectories that evolution could follow [15]. This may in part account for the commonly observed instances of parallel evolution [33], [93]–[95].

With this survey, we established several trends of functional conservation and divergence of *cis*-regulatory elements. We found pervasive functional divergence in transcriptional regulatory mechanisms, both in *cis* and in *trans*. More strikingly, we identified inherent biases in the nature and functional consequences of this divergence, hinting at possible mechanisms underlying repeated evolution.

## Materials and Methods

### Cloning of *cis*-regulatory elements

Putative *cis*-regulatory elements (extending from the first exon to the nearest upstream gene) were PCR amplified from genomic DNA using Phusion polymerase and cloned upstream of GFP into the pPD95.75 plasmid, routinely used for analysis of gene expression in *C. elegans* [96]. Cloned fragments were sequenced to ensure accuracy. *C. elegans* CREs of *unc-46*, *acr-14*, *kat-1*, *unc-25*, and *oig-1*, were also cloned into the plasmid HYM153 (kind gift of H.-Y. Mak) upstream of the mCherry reporter gene as controls.

### Strains

*C. elegans* transgenic lines were established by injecting into *pha-1(e2123)* worms cocktails consisting of 5 ng/µL CRE::GFP reporter constructs with 5 ng/µL rescue plasmid [97] and 100 ng/µL salmon sperm DNA; this is thought to facilitate the formation of complex transgenic constructs as extrachromosomal arrays [98]. For five genes (*unc-46*, *acr-14*, *kat-1*, *unc-25*, and *oig-1*), plasmids carrying *C. elegans* CREs fused to mCherry were coinjected with the plasmids carrying orthologous CREs from each of the five species fused to GFP. *C. briggsae* transgenic lines were established by injecting cocktails consisting of 5 ng/µL CRE::GFP reporter constructs with 5 ng/µL rescue plasmid and 100 ng/µL salmon sperm DNA into *Cbr-unc-119 (nm67)* worms [99].

### Microscopy

Mixed-stage populations of transgenic worms were grown with abundant food and L4-stage larvae or young adults were selected. These were immobilized on agar slides with 10 mM sodium azide in M9 buffer. The slides were examined on a Leica DM5000B compound microscope under 400-fold magnification. Worms without any visible GFP expression were assumed to have lost the transgene. Each photograph showing worms in figures is composed of several images of the same individual capturing anterior, middle, and posterior sections.

### Analysis

At least fifty individuals from no fewer than two independent strains were analyzed for each transgene. The plasmid pPD95.75 has been used extensively by the *C. elegans* community over the last two decades. It has been reported to direct low-level background expression in the pharynx and anterior and posterior intestine [49], [92], [96]. We have previously reported that extrachromosomal arrays direct expression patterns that are concordant with those of integrated and single-copy transgenes [22], [100]. Still, to obtain conservative estimates of expression differences between CREs from *C. elegans* and other species, we only counted discrepancies (missing or extra expression) observed in two or more strains. Data on consistency of expression patterns between strains and individuals are presented in Tables S1, S2, S3, S4, S5, S6, S7, S8 and S10.

## Supporting Information

Figure S1 Synteny is conserved across all five species for the eight genes studied. Schematic representation of synteny and intergenic distances for *unc-46*, *acr-14*, *unc-47*, *kat-1*, *unc-25*, *gpa-5*, *oig-1*, and *mod-5*. In each set, from top to bottom: *C. elegans*, *C. briggsae*, *C. remanei*, *C. brenneri*, *C. japonica*.
(PDF)

Figure S2 Divergence in *trans*-regulatory mechanisms. (A–D) Comparisons of the expression patterns driven in *C. elegans* and *C. briggsae* by CREs of (A) *C. briggsae unc-46*, (B) *C. briggsae unc-25*, (C) *C. briggsae gpa-5*, (D) *C. briggsae oig-1*. Abbreviations of cell names and the meaning of values are the same as in corresponding Figures 2B, 6B, 7B, and 8B. Detailed data are shown in Table S11.
(PDF)

Figure S3 Functional divergence does not correlate with complexity of expression patterns or primary sequence conservation. (A) Complexity of expression pattern, measured as the number of endogenously expressing cell types, does not correlate with functional divergence of *cis*-regulatory elements, as measured by differences (expressing cell types) of *C. elegans* and orthologous CREs. (B) Primary sequence conservation, as measured by the fraction of CRE sequences contained in conserved blocks of 20 nucleotides or more, does not correlate with functional divergence of *cis*-regulatory elements. (C) Primary sequence conservation does not correlate with complexity of expression patterns. (D) Difference in length of CRE sequences does not correlate with functional divergence. Each data point represents a single *cis*-regulatory element; all comparisons are to *C. elegans*.
(PDF)

Figure S4 “Gains” of expression are more frequent than “losses.” The curves represent sorted frequencies of “losses” of expression along the endogenous pattern (blue) and “gains” of expression (pink). Frequency of “loss” refers to frequency of endogenous cells not expressing a heterologous transgene. Frequency of “gain” refers to frequency of expression in non-endogenous cells. For example, a frequency of 20% “loss” refers to 80% of transgenic individuals showing expression in a particular cell type, whereas 20% “gain” indicates that 20% of transgenic individuals show ectopic expression in a particular cell type. Since expression in the ventral nerve cord was measured as a median, and not a frequency, this plot does not include ventral nerve cord data. For every possible frequency threshold below 100%, instances of “gain” outnumber instances of “loss.”
(PDF)

Table S1 Conservation of primary sequence in CREs between *C. elegans* and *C. briggsae*.
(XLSX)

Table S2 “Gains” and “losses” of expression relative to *C. elegans*.
(DOC)

Table S3 Expression patterns of *unc-46 cis* elements. Raw data for expression patterns reported in Figure 2.
(XLS)

Table S4 Expression patterns of *acr-14 cis* elements. Raw data for expression patterns reported in Figure 3.
(XLS)

Table S5 Expression patterns of *unc-47 cis* elements. Raw data for expression patterns reported in Figure 4.
(XLS)

Table S6 Expression patterns of *kat-1 cis* elements. Raw data for expression patterns reported in Figure 5. Expression is counted as present or absent in a specific cell type.
(XLS)

Table S7 Expression patterns of *unc-25 cis* elements. Raw data for expression patterns reported in Figure 6.
(XLS)

Table S8 Expression patterns of *gpa-5 cis* elements. Raw data for expression patterns reported in Figure 7.
(XLS)

Table S9 Expression patterns of *oig-1 cis* elements. Raw data for expression patterns reported in Figure 8.
(XLS)

Table S10 Expression patterns of *mod-5 cis* elements. Raw data for expression patterns reported in Figure 9.
(XLS)

Table S11 Expression patterns of *C. briggsae* transgenes in *C. briggsae*. Raw data for expression patterns reported in Figure S2.
(XLS)

Text S1 Detailed description of Figures 2–9 and supplemental discussion.
(DOC)
