# Supplementary figures and images for: Pervasive Divergence of Transcriptional Gene Regulation in Caenorhabditis Nematodes
Source: PLoS Genet. 2014 Jun 26;10(6):e1004435. doi: 10.1371/journal.pgen.1004435 (PMC4072541; doi:10.1371/journal.pgen.1004435)

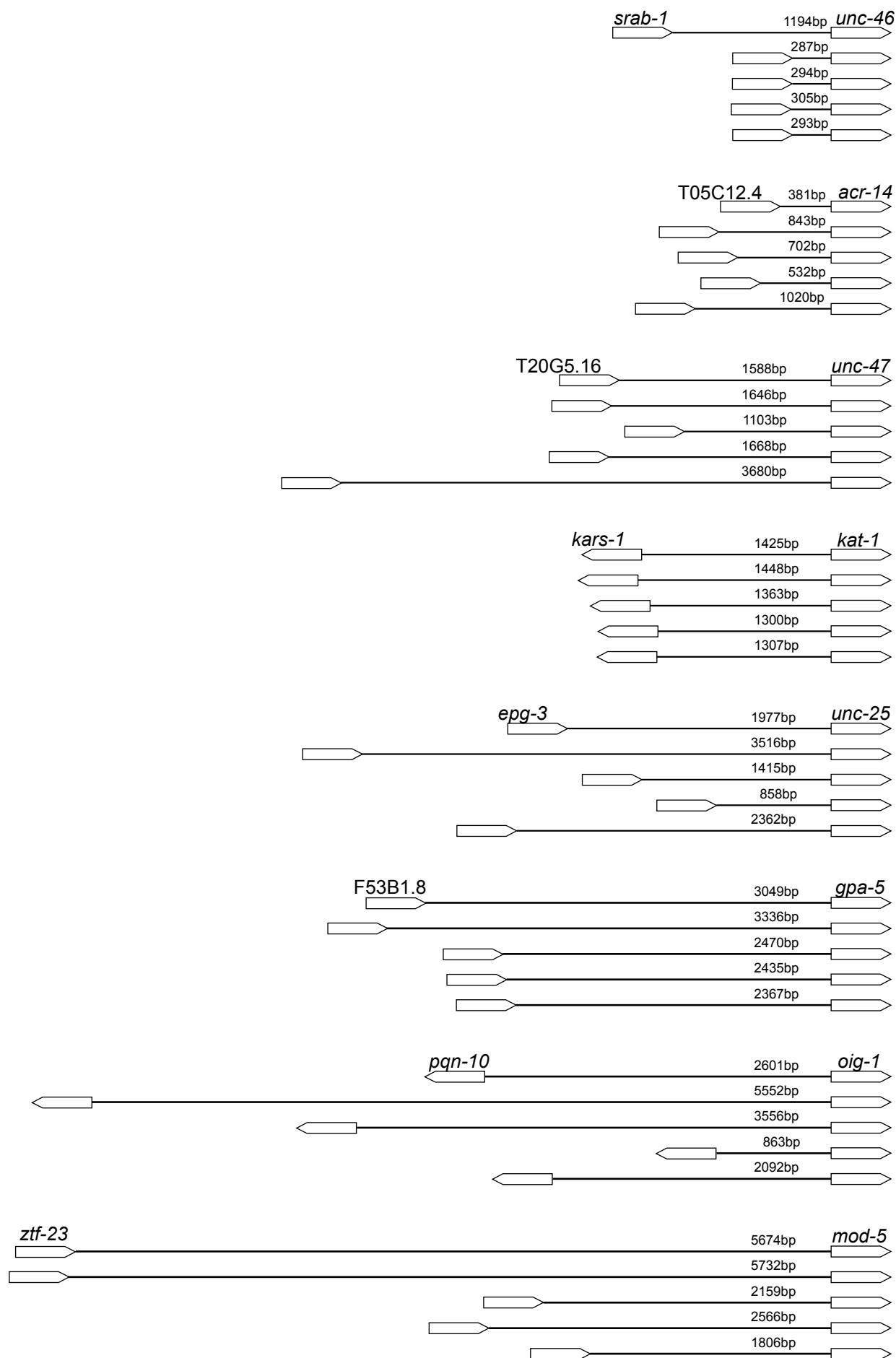

Figure S1

Supplement: Figure S1 — Synteny is conserved across all five species for the eight genes studied. Schematic representation of synteny and intergenic distances for unc-46, acr-14, unc-47, kat-1, unc-25, gpa-5, oig-1, and mod-5. In each set, from top to bottom: C. elegans, C. briggsae, C. remanei, C. brenneri, C. japonica. (PDF) [file pgen.1004435.s001.pdf]

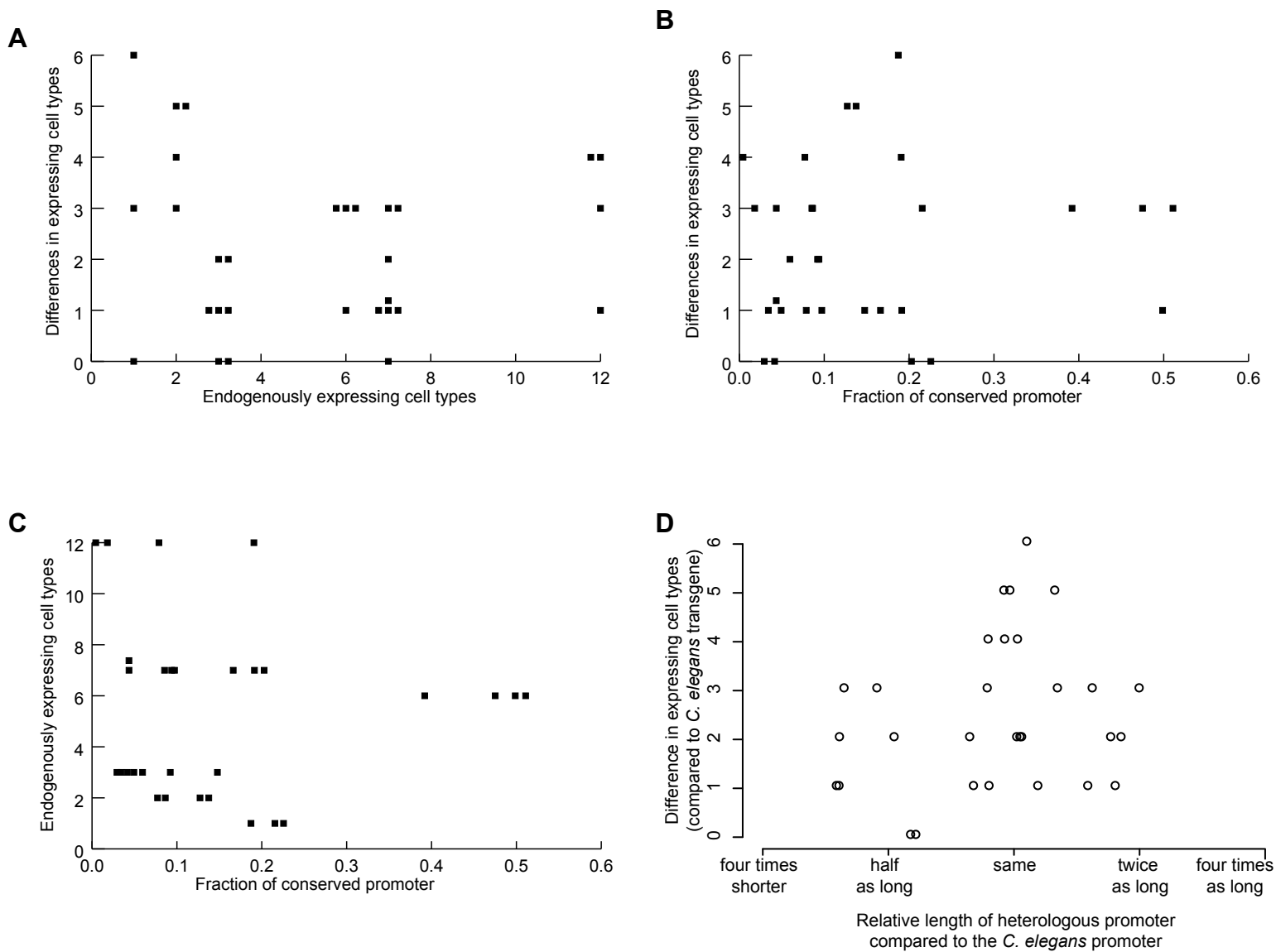

**Figure S3**

Supplement: Figure S3 — Functional divergence does not correlate with complexity of expression patterns or primary sequence conservation. (A) Complexity of expression pattern, measured as the number of endogenously expressing cell types, does not correlate with functional divergence of cis-regulatory elements, as measured by differences (expressing cell types) of C. elegans and orthologous CREs. (B) Primary sequence conservation, as measured by the fraction of CRE sequences contained in conserved blocks of 20 nucleotides or more, does not correlate with functional divergence of cis-regulatory elements. (C) Primary sequence conservation does not correlate with complexity of expression patterns. (D) Difference in length of CRE sequences does not correlate with functional divergence. Each data point represents a single cis-regulatory element; all comparisons are to C. elegans. (PDF) [file pgen.1004435.s003.pdf]

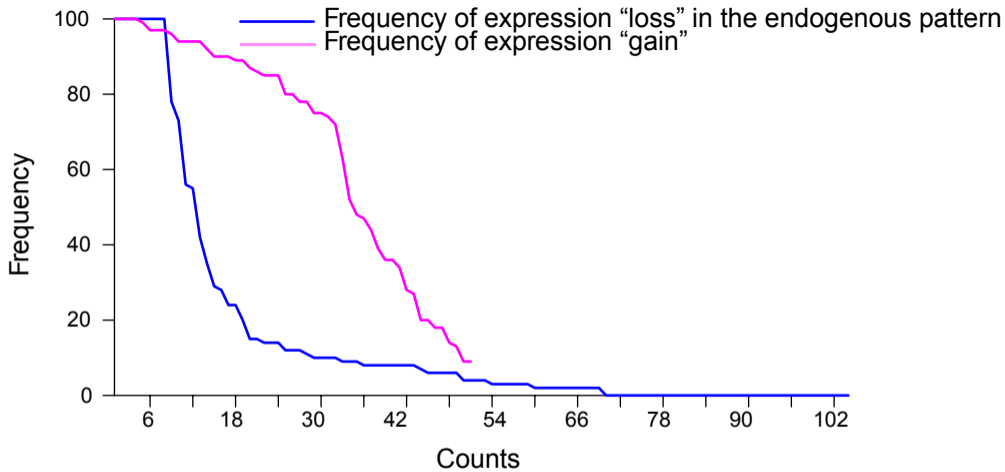

**Figure S4**

Supplement: Figure S4 — “Gains” of expression are more frequent than “losses.” The curves represent sorted frequencies of “losses” of expression along the endogenous pattern (blue) and “gains” of expression (pink). Frequency of “loss” refers to frequency of endogenous cells not expressing a heterologous transgene. Frequency of “gain” refers to frequency of expression in non-endogenous cells. For example, a frequency of 20% “loss” refers to 80% of transgenic individuals showing expression in a particular cell type, whereas 20% “gain” indicates that 20% of transgenic individuals show ectopic expression in a particular cell type. Since expression in the ventral nerve cord was measured as a median, and not a frequency, this plot does not include ventral nerve cord data. For every possible frequency threshold below 100%, instances of “gain” outnumber instances of “loss.” (PDF) [file pgen.1004435.s004.pdf]
