# Supplementary material for: Pervasive Divergence of Transcriptional Gene Regulation in Caenorhabditis Nematodes
Source: PLoS Genet. 2014 Jun 26;10(6):e1004435. doi: 10.1371/journal.pgen.1004435 (PMC4072541; doi:10.1371/journal.pgen.1004435)
Supplement: Figure S2 — Divergence in trans-regulatory mechanisms. (A–D) Comparisons of the expression patterns driven in C. elegans and C. briggsae by CREs of (A) C. briggsae unc-46, (B) C. briggsae unc-25, (C) C. briggsae gpa-5, (D) C. briggsae oig-1. Abbreviations of cell names and the meaning of values are the same as in corresponding Figures 2B, 6B, 7B, and 8B. Detailed data are shown in Table S11. (PDF) [file pgen.1004435.s002.pdf]

A

unc-46

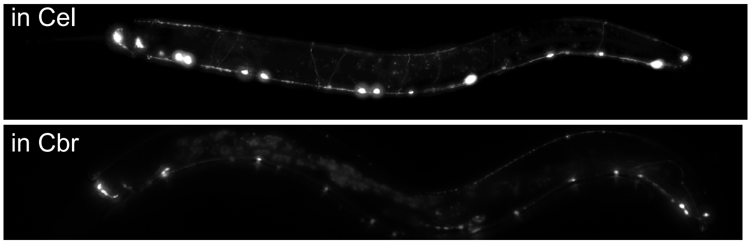

	RMEs	AVL	SIADs	RIS	D-type (median)	DVB	Lat. gang.	PDB
in Cel	100%	88%	96%	90%	18	86%	34%	
in Cbr	97%	75%	96%	28%	18.5	85%		72%

B

unc-25

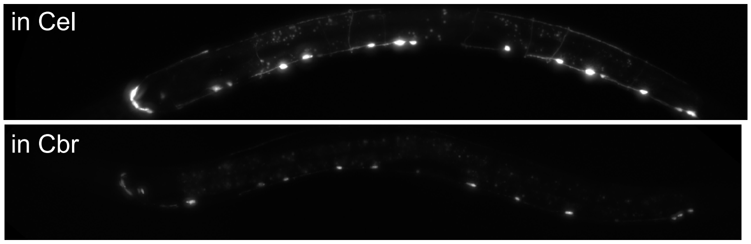

	RMEs	AVL	RIS	D-type (median)	SIADs
in Cel	100%			16	52%
in Cbr	100%	12% (weak)	41% (weak)	15	100%

C

gpa-5

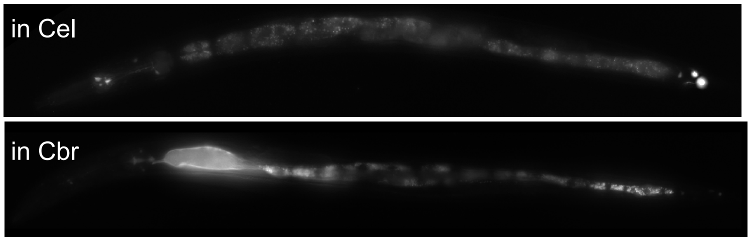

	AWAs	MCs	M4	M1	DVB	PVNs	PHCs
in Cel	45% (weak)	100%	85%	85%	87%	94%	
in Cbr	47% (weak)	89% (weak)	21% (weak)	79% (weak)	66% (weak)		95% (weak)

D

oig-1

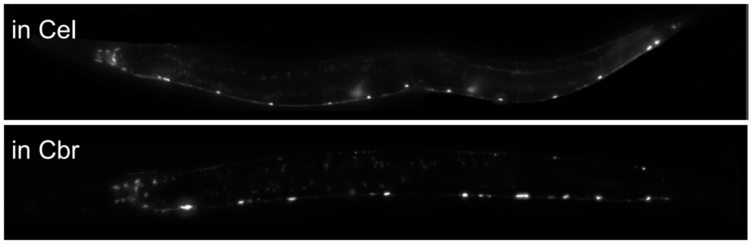

	Head neurons	D-type (median)	DVB	PVCs	HSNs	PDEs
in Cel	100%	14	72%	100%	36%	
in Cbr	100%	17		63% (weak)		29%

Figure S2
