# Supplementary material for: Pervasive Divergence of Transcriptional Gene Regulation in Caenorhabditis Nematodes
Source: PLoS Genet. 2014 Jun 26;10(6):e1004435. doi: 10.1371/journal.pgen.1004435 (PMC4072541; doi:10.1371/journal.pgen.1004435)
Supplement: Table S2 — “Gains” and “losses” of expression relative to C. elegans. (DOC) [file pgen.1004435.s006.doc]

|  | Gains | Losses (high stringency) | Losses (low stringency)* |
| --- | --- | --- | --- |
| *C. briggsae* | 9 | 1 | 2 |
| *C. remanei* | 13 | 4 | 5 |
| *C. brenneri* | 11† | 4 | 5 |
| *C. japonica* | 11 | 3‡ | 4 |

**Table S2.** Gains and losses relative to *C. elegans*

* Counted as weak reductions, and thus in Losses (low stringency), are: *C. brenneri unc-47* in AVL, *C. japonica kat-1* in the gonadal sheath, *C. briggsae oig-1* in DVB, and *C. remanei oig-1* in the PVCs.

† Given that the *C. elegans* *unc-47* CRE expresses weakly in SDQR, the weak expression in the SDQs driven by the *C. brenneri* *unc-47* CRE is not counted as a gain.

‡ Since the *C. japonica acr-14* CRE fails to drive expression in two ventral cord neuronal classes, ASs and DBs, this event is counted as two losses.
