# Supplementary material for: Pervasive Divergence of Transcriptional Gene Regulation in Caenorhabditis Nematodes
Source: PLoS Genet. 2014 Jun 26;10(6):e1004435. doi: 10.1371/journal.pgen.1004435 (PMC4072541; doi:10.1371/journal.pgen.1004435)
Supplement: Text S1 — Detailed description of Figures 2–9 and supplemental discussion. (DOC) [file pgen.1004435.s016.doc]

**Text S1**

*unc-46*

The *unc-46* gene encodes a protein required for the trafficking of the vesicular GABA transporter and is expressed in all GABA neurons in *C. elegans* [1]. Its expression in D-type neurons is controlled by the transcription factor UNC-30 [2]. The *C. elegans* *cis* element of *unc-46* consistently drove expression in the 26 GABAergic neurons: four RMEs, AVL, RIS, DVB, and 19 D-type neurons in the ventral nerve cord (Figure 2B). In addition, we observed a previously unreported expression in a pair of cells in the lateral ganglion, tentatively identified as neurons SIADL/R. The expression pattern driven by the *C. elegans* *cis* element was recapitulated by the CREs from *C. briggsae*, *C. remanei*, *C. brenneri*, and *C. japonica* when introduced into *C. elegans*. In addition, all of these exogenous *cis* elements drove some ectopic expression in *C. elegans* neurons: *C. remanei* and *C. brenneri* CREs in HSNL/R, the *C. brenneri* CRE in OLQDL/R and all four in an unidentified pair of neurons in the lateral ganglion in the head.

*acr-14*

The *acr-14* gene encodes an acetylcholine receptor and is expressed in D-type and other ventral cord neurons, as well as some head neurons [3]. Its expression in D-type neurons is controlled by the transcription factor UNC-30 [2]. The *C. elegans cis* element of *acr-14* directed expression in one pair of cells in the lateral ganglion in the head, AVAL/R, and neurons (median count was 41) in the ventral cord (Figure 3B). Based on the number of cells and the morphology of their processes, we identified these neurons as DAs, DDs, and VDs, with some contribution from the DB and AS classes. The *C. briggsae*, *C. remanei*, and *C. brenneri* *cis* elements directed expression in AVAL/R at the same frequency and intensity as *C. elegans*, whereas the *C. japonica* CRE was expressed at a significantly lower frequency (binomial test, *p*=3.3﻿×10^-9^) and intensity. Expression of *C. briggsae* and *C. remanei* CREs in the ventral nerve cord was substantially conserved, while the *C. brenneri* CRE was expressed in fewer cells. Expression of the *C. japonica* CRE was restricted to the D-type neurons – DDs and VDs. Exogenous *cis* elements also directed ectopic neuronal expression. All shared the ability to express in a pair of lateral ganglion neurons in the head, the identity of which was narrowed down to AVH, AVJ or AVDL/R (here referred to as AVnL/R), although the *C. japonica* CRE showed inconsistent expression. In addition, the *C. remanei* *cis* element was active in AFDL/R and PVNL/R, while the *C. brenneri* one drove expression in neurons tentatively identified as CEPD/V/L/R.

*unc-47*

The *unc-47* gene encodes the vesicular GABA transporter that functions in all GABAergic neurons in *C. elegans* [4]. Its expression in D-type neurons is controlled by the transcription factor UNC-30 [5]. In *C. elegans,* the endogenous pattern of expression has been described as the 26 GABAergic neurons: four RMEs, AVL, RIS, DVB, and 19 D-type neurons in the ventral nerve cord [5]. Similarly to *unc-46* (see above), we observed a previously unreported expression in a pair of lateral ganglion cells in the head, tentatively identified as SIADL/R (Figure 4B). When placed in *C. elegans*, *cis* elements of the four orthologs recapitulated the majority of this pattern: the four RMEs, AVL, DVB and the D-type neurons in the ventral cord. As reported previously, the levels were comparable to those of the *C. elegans* CRE [6], except for the *C. remanei* element, which was expressed weaker and significantly less frequently in RIS (binomial test, *p*=8.8﻿×10^-4^). Expression in the SIAD neurons was only directed by the *C. japonica*, not *C. briggsae*, *C. remanei* or *C. brenneri* CREs. As reported previously [6], the *C. briggsae* element drives strong and consistent expression in SDQR/L, same two neurons in which weak and inconsistent expression of the *C. brenneri* CRE is also seen. This latter *cis* element was active in the intestine (at levels that were much brighter than the background commonly seen with GFP transgenes), as well as in several unidentified head neurons. Finally, the *C. japonica* CRE directed expression in four CEP neurons in the head and PVNL/R in the tail.

*kat-1*

The *kat-1* gene encodes an enzyme in the mitochondrial oxidation pathway that was reported to be expressed in the pharynx, intestine, and body wall muscle [7]. The *C. elegans* *cis* element we used was active in the pharynx, intestine, and proximal gonadal sheath, but not in the body wall muscle (Figure 5B). All orthologous CREs recapitulated the pharyngeal and intestinal expression, but those of *C. remanei* and *C. brenneri* did not appear to direct expression in the gonadal sheath. However, there was ectopic expression in multiple cell types: in head muscles (only the *C. briggsae* and *C. brenneri* ones did so consistently), the hypodermis, several head neurons, approximately a dozen ventral cord neurons (*C. remanei*), and some phasmid sheath cells.

*unc-25*

The *unc-25* gene encodes the glutamic acid decarboxylase, an enzyme that synthesizes GABA, and is expressed in all GABAergic neurons [8]. Its expression in D-type neurons is controlled by the transcription factor UNC-30 [2]. Consistent with previous reports [5,9], the intergenic region alone drove expression only in the four RME head neurons and the D-type neurons in the ventral cord (Figure 6B). All four exogenous *cis* elements recapitulated this pattern in *C. elegans*. In addition, the CREs from *C. briggsae*, *C. remanei*, and *C. japonica*, but not *C. brenneri*, directed inconsistent expression in the SIAD neurons (compare with *unc-46* and *unc-47*). To rule out the possibility that conserved expression is mediated by extended stretches of conserved sequence (equivalent to those located between approximately -350-600bp in the *C. elegans cis* element), we tested shorter promoters that only contained the sequences up to these conserved blocks (data not shown). Consistent with previous reports [8,9], these shorter elements were sufficient to recapitulate expression patterns generated by the entire intergenic sequences.

*gpa-5*

The *gpa-5* gene encodes a subunit of GTPase that was reported to be expressed in the AWA neurons and, faintly, in the neighboring ASI neurons [10]. The pattern we observed with the *C. elegans cis* element is consistent with this report (Figure 7B). Exogenous CREs were capable of expression in the AWAs, although the *C. briggsae* and *C. brenneri* ones did so less consistently, but no expression was seen in the ASIs. Strong and consistent ectopic expression was seen in multiple neurons in the head and tail. We also tested shorter constructs that only contained the sequences between translation start sites and the blocks of substantial conservation located upstream of ~1.3kb (data not shown). In all examined instances, shorter elements recapitulated the patterns generated by their longer counterparts, albeit weakly.

*oig-1*

The *C. elegans* *cis* element of *oig-1* was expressed in multiple anterior neurons, including ALAL/R, SMDVL/R, RMDVL/R, RIAL/R, AVAL/R, RIML/R, RMDDL/R and IL1s (we refer to them collectively as head neurons), as well as D-type neurons in the ventral cord (median number was 15), DVB and PVCL/R in the tail (Figure 8B). Expression in D-type neurons is controlled by the transcription factor UNC-30 [2]. Some expression was seen in the spermatheca. This pattern was substantially recapitulated by the CREs of other species, except for a significant reduction in the frequency of expression in DVB by the *C. remanei*, *C. brenneri*, and *C. japonica* CREs. Due to the density of expression in the head, some minor differences in expression pattern may have been missed. All four exogenous CREs showed ectopic neuronal expression – *C. remanei*, *C. brenneri*, and *C. japonica* in ADEL/R and PDEL/R, and the *C. briggsae*, *C. remanei*, and *C. japonica*, although less consistently, in HSNL/R.

*mod-5*

The *mod-5* gene encodes a serotonin transporter expressed in all serotonergic neurons [11]. The intergenic sequence upstream of *C. elegans mod-5* drove expression in a subset of these cells: ADFL/R, RIH, and AIML/R (Figure 9B). Expression in only one pair of these neurons (ADFL/R) was seen with all exogenous *cis* elements. Only the *C. brenneri* CRE appeared to be functionally equivalent to its *C. elegans* counterpart. We did not observe any ectopic expression.

Plausible cause of repeated observation of ectopic expression in the same neurons

We attempted to establish whether it is likely that the cells (namely, CEPs, HSNs, and PVNs) showing ectopic expression of different constructs are “promiscuously” expressing cells. If they were, it might be expected that expression of multiple other transgenes would have been reported in these cells by other researchers. We counted the number of genes (as recorded in wormbase.org) reported as being expressed in these three cell types (we only considered expression patterns labeled as “certain”). Expression in CEPs, HSNs, and PVNs was reported for 20, 77, and 5 genes respectively. We attribute the high number of HSN-containing reports to these being easily identifiable neurons that were studied extensively due to a role in egg-laying. Even so, these numbers do not appear particularly high compared, for example, to the number of genes (50) reported to be expressed in DD neurons (where *acr-14*, *oig-1*, *unc-46*, and *unc-47* are endogenously expressed). For this reason, we feel that it is more plausible that repeated observation of ectopic expression in CEPs, HSNs, and PVNs is better explained by bias rather than “promiscuous” nature of these cells.

**References**

1. Schuske K, Palfreyman MT, Watanabe S, Jorgensen EM (2007) UNC-46 is required for trafficking of the vesicular GABA transporter. Nat Neurosci 10: 846–853. doi:10.1038/nn1920.

2. Cinar H, Keles S, Jin Y (2005) Expression profiling of GABAergic motor neurons in Caenorhabditis elegans. Curr Biol 15: 340–346. doi:10.1016/j.cub.2005.02.025.

3. Fox RM, Von Stetina SE, Barlow SJ, Shaffer C, Olszewski KL, et al. (2005) A gene expression fingerprint of C. elegans embryonic motor neurons. BMC Genomics 6: 42. doi:10.1186/1471-2164-6-42.

4. McIntire SL, Reimer RJ, Schuske K, Edwards RH, Jorgensen EM (1997) Identification and characterization of the vesicular GABA transporter. Nature 389: 870–876. doi:10.1038/39908.

5. Eastman C, Horvitz HR, Jin Y (1999) Coordinated transcriptional regulation of the unc-25 glutamic acid decarboxylase and the unc-47 GABA vesicular transporter by the Caenorhabditis elegans UNC-30 homeodomain protein. J Neurosci 19: 6225–6234.

6. Barrière A, Gordon KL, Ruvinsky I (2012) Coevolution within and between Regulatory Loci Can Preserve Promoter Function Despite Evolutionary Rate Acceleration. PLoS Genet 8: e1002961. doi:10.1371/journal.pgen.1002961.

7. Mak HY, Nelson LS, Basson M, Johnson CD, Ruvkun G (2006) Polygenic control of Caenorhabditis elegans fat storage. Nat Genet 38: 363–368. doi:10.1038/ng1739.

8. Jin Y, Jorgensen EM, Hartwieg E, Horvitz HR (1999) The Caenorhabditis elegans gene unc-25 encodes glutamic acid decarboxylase and is required for synaptic transmission but not synaptic development. J Neurosci 19: 539–548.

9. Ruvinsky I, Ruvkun G (2003) Functional tests of enhancer conservation between distantly related species. Development 130: 5133–5142. doi:10.1242/dev.00711.

10. Jansen G, Thijssen K, Werner P (1999) The complete family of genes encoding G proteins of Caenorhabditis elegans. Nat Genet 21: 414–419.

11. Jafari G, Xie Y, Kullyev A, Liang B, Sze JY (2011) Regulation of extrasynaptic 5-HT by serotonin reuptake transporter function in 5-HT-absorbing neurons underscores adaptation behavior in Caenorhabditis elegans. J Neurosci 31: 8948–8957. doi:10.1523/JNEUROSCI.1692-11.2011.
